# Development of an *ex vivo* preclinical respiratory model of idiopathic pulmonary fibrosis for aerosol regional studies

**DOI:** 10.1038/s41598-019-54479-2

**Published:** 2019-11-29

**Authors:** Yoann Montigaud, Sophie Périnel-Ragey, Laurent Plantier, Lara Leclerc, Clémence Goy, Anthony Clotagatide, Nathalie Prévôt, Jérémie Pourchez

**Affiliations:** 1 Mines Saint-Etienne, Univ Lyon, Univ Jean Monnet, INSERM, U 1059 Sainbiose, Centre CIS, F - 42023 Saint-Etienne, France; 2 INSERM U 1059 Sainbiose, Université Jean Monnet, Saint-Etienne, France; 3 CHU Saint-Etienne, Saint-Etienne, F-42055 France; 4 CEPR/INSERM UMR1100, LabexMabImprove & Service de Pneumologie et Explorations Fonctionnelles Respiratoires, Hôpital Bretonneau, Université François Rabelais, Tours, F-37044 France

**Keywords:** Preclinical research, Physiology, Respiratory tract diseases

## Abstract

Idiopathic pulmonary fibrosis is a progressive disease with unsatisfactory systemic treatments. Aerosol drug delivery to the lungs is expected to be an interesting route of administration. However, due to the alterations of lung compliance caused by fibrosis, local delivery remains challenging. This work aimed to develop a practical, relevant and ethically less restricted *ex vivo* respiratory model of fibrotic lung for regional aerosol deposition studies. This model is composed of an Ear-Nose-Throat replica connected to a sealed enclosure containing an *ex vivo* porcine respiratory tract, which was modified to mimic the mechanical properties of fibrotic lung parenchyma - *i.e*. reduced compliance. Passive respiratory mechanics were measured. ^81m^Kr scintigraphies were used to assess the homogeneity of gas-ventilation, while regional aerosol deposition was assessed with ^99m^Tc-DTPA scintigraphies. We validated the procedure to induce modifications of lung parenchyma to obtain aimed variation of compliance. Compared to the healthy model, lung respiratory mechanics were modified to the same extent as IPF-suffering patients. ^81m^Kr gas-ventilation and ^99m^Tc-DTPA regional aerosol deposition showed results comparable to clinical studies, qualitatively. This *ex vivo* respiratory model could simulate lung fibrosis for aerosol regional deposition studies giving an interesting alternative to animal experiments, accelerating and facilitating preclinical studies before clinical trials.

## Introduction

Idiopathic pulmonary fibrosis (IPF), a progressive fibrotic disease of the lungs without identified etiology, is the most common form of idiopathic interstitial pneumonia^1,2^. Estimated incidence is around 2.8/100000 in North America and Europe, while lower incidences are observed in Asia and South America^3^. The spontaneous 3–5 years survival is around 50%^4–6^. IPF is characterized by progressive fibrotic lesions extending into the lungs from subpleural regions with a heterogeneous distribution throughout the lung. This leads to impairments of lung mechanics with a prominent reduction of lung compliance^7–12^ - *i.e* decreased ability of the lung to stretch and expand during the breathing cycle. IPF symptoms include cough, exertional dyspnea^1,7,13–18^, alterations in pulmonary gas exchange^8,17^, physiology of airways^19^ and pulmonary hemodynamics^14–16,20,21^.

Currently, IPF is treated with systemic antifibrotic drugs, such as pirfenidone and nintedatinib, which have been shown to delay the progressive decrease of lung function and to reduce mortality^3,22–24^. However, neither pirfenidone nor nintedatinib stops disease progression, while lung transplantation is associated with significant morbidity and mortality^25–28^. Thus, new treatments for IPF are strongly needed.

Pulmonary delivery of drugs is expected to be an interesting route of administration as an alternative to systemic therapies in IPF. Indeed, work is ongoing to develop inhaled IPF therapies using either repurposed drugs such as interferon gamma^29^ or new chemical entities, such as the α_v_β_6_ integrin inhibitor GSK3008348^30^. Nevertheless, optimization of nebulization technologies appears necessary to reach this aim. Indeed, due to the alterations of lung compliance in IPF, aerosolized delivery of treatments remains challenging. Heterogeneous reduction of lung compliance is associated with impaired deposition of aerosol in affected pulmonary regions^31^. Moreover, human *in vivo* data lacks of precise mapping and quantification of aerosol deposition patterns in IPF. Thus, there is an urgent need for inexpensive preclinical studies devoted to the quantitative assessment of aerosol regional deposition in fibrotic lungs. Such studies would help to improve nebulization technologies for local administration to fulfill the unmet need for better treatments.

Airborne particles properties (*e.g*. aerodynamic size, hydroscopic properties, electrical surface charge, etc.) are some of the main parameters affecting regional deposition of aerosolized drugs within the respiratory tract. However, anatomical and physiological features (*e.g*. diameter and branching of airways, breathing frequency, tidal volume, etc.)^32,33^ also represent key factors in regional deposition. Although they allow to accurately study these parameters, *in vivo* human studies are scarce due to ethical considerations and costs involved, as radiolabeled aerosols are the gold standard method to assess regional deposition of inhaled particles within lungs^34^. Consequently, animals (particularly rodents) are common surrogate models even if animal use also suffers from ethical restrictions^35,36^. Animal models are also time-consuming and expensive due to costs involved in breeding and housing. Moreover, such models are different from humans in terms of anatomy of upper airways, bronchial divisions and lung segmentation^37^. Ventilation patterns are also very different in rodents. As an example, breathing frequency at rest for rats is about 80 cycles per minute while it is 15 per minute for adult humans. Additionally, no model sufficiently reproduces the pathophysiological features of IPF lungs that it may be used for relevant aerosol deposition studies. These discrepancies are commonly recognized as biases in aerosol deposition patterns. Thus, extrapolation from these models to humans should be carefully performed. To overcome these limitations, mathematical and computational deposition models for inhaled particles were developed but were mainly based on extrapolation from healthy young adults^38–40^. Moreover, these models are confronted to a data gap for some parameters, such as submicron sized particles or specific anatomical features. This lack of database is challenging for these models and lead to issues to validate obtained predictions. Consequently, inhalation studies require relevant and reliable respiratory models.

Therefore, based on previous works of our laboratory^41,42^, this study aimed to develop an adult *ex vivo* respiratory model, which could be a useful tool for practical and less expensive preclinical studies devoted to the local delivery of drugs to fibrotic lungs. This respiratory model is composed of a 3D-printed Ear-Nose-Throat (ENT) replica connected to an *ex vivo* porcine respiratory tract placed in a sealed instrumented enclosure. To mimic some of the alterations associated with fibrosis, lungs were modified to reduce the lung compliance and to create restrictive abnormalities at lung bases and upper lobes representing fibrotic lesions. Then, lungs were ventilated by applying negative pressure in the enclosure to simulate pleural depression and, thus, passive ventilation. Aims of this work were to validate the reliability of this innovative *ex vivo* model of pulmonary fibrosis and to compare it, in terms of respiratory mechanics and features of aerosol regional deposition, to the previously developed healthy *ex vivo* respiratory model^41,42^ (*i.e*. before the modification performed to depict the physiological alterations associated with IPF) and to IPF-suffering patients^7,8,10,12,19,20,31,43–45^. Consequently, this study is divided into 4 main parts: (i) development and comparison of various physical procedures to reduce the pulmonary compliance (ii) measurements of respiratory mechanics before and after modifications of lung tissue, (iii) assessment of regional gas-ventilation and (iv) regional aerosol deposition within fibrotic lungs.

## Results and Discussion

### Performance analysis of physical methods to reduce lung compliance mimicking the mechanical behavior of a fibrotic lung

**Figure 1:**
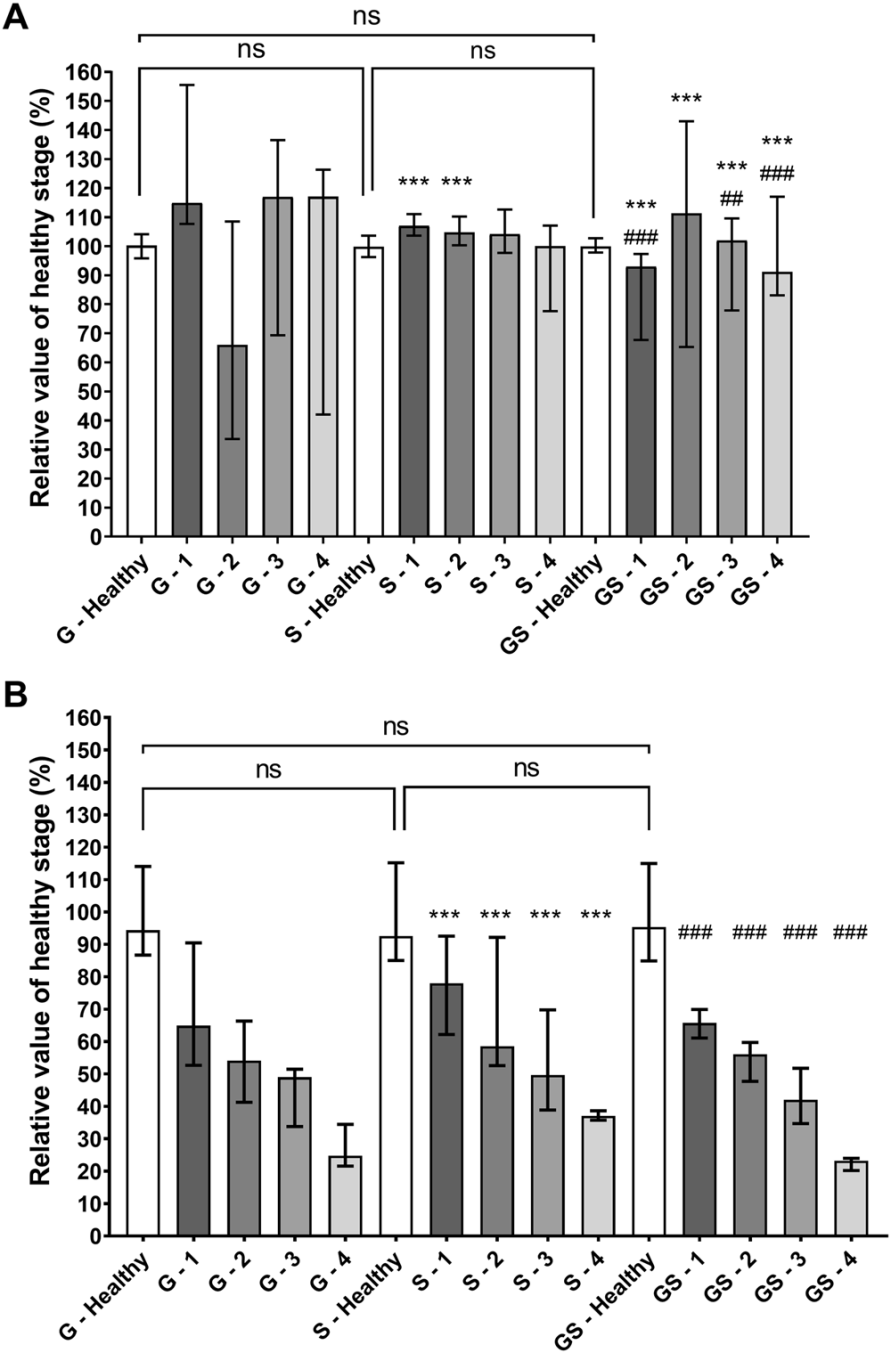
Variability of resistances (**A**) and compliance (**B**) with different fibrosis methods and stages (n = 4). G: Glue method. S: Steam method. GS: Glue + Steam method. 1: fibrosis of apices of upper lobe; 2: fibrosis of entire upper lobes; 3: fibrosis of entire upper lobes + bases of lower lobes; 4: fibrosis of entire upper lobes + entire lower lobes. “G healthy” (respectively the “S healthy” and the “GS healthy”) corresponds to the lungs before modifications which will be induced at different stages by the G method (respectively the S method and the GS method). Data are presented as median and inter-quartile range of the relative value of the healthy stage of the corresponding method. ***Corresponds to p < 0.0001 when compared to glue method. ## and ### corresponds to p < 0.001 and p < 0.0001 respectively when compared to steam method. ns corresponds to non-significant.

These experiments aimed to find a reliable and reproducible method to simulate lung fibrosis on *ex vivo* porcine respiratory tracts (Fig. 1 and Tables S1 and S2 the Supplementary Information). Figure 1 presents the resistances and the dynamic compliance of the respiratory tract expressed as mean ± standard deviation of the relative value of the “healthy” stage of the lungs (*i.e*. lungs with no modification). Resistances (R) with glue (G) method appeared to be uneven with important dispersion of data. Inversely, steam (S) method showed a much narrower dispersion and a slight modification of R when compared to the healthy stage. However, when reaching stage 4 (*i.e*. methods applied to whole lungs) with S method we observed an important increase of the standard deviation showing a lack of reproducibility for this specific stage (see Supplementary Information). Glue + steam (GS) method suffered from a wide dispersion of data as G method (Fig. 1A). Dynamic compliance of lung decreased with the incrementing stages of the three methods. The strongest variations of compliance were seen with G and GS method. S method induced a less important reduction of compliance (Fig. 1B).

Considering the presented results, G and GS methods were generating barely reproducible data, while S method was more reproducible and repeatable. One hypothesis explaining the differences between G and S methods is that G method is more likely to simulate a pleural disease while the thermal treatment of the S method is more likely to simulate “fibrotic lung pattern” by denaturing extracellular matrix proteins^46–48^. Moreover, applying the S method on lung parenchyma induced a visual shrinking of the treated regions. However, we did not assess that this thermal treatment had any significant impact of the small airways, which exert a relative widening in parenchymal regions suffering from IPF^19,21^. Thus, careful extrapolation should be made for this point, which should not impact the validity of aerosol deposition pattern. Therefore, we decided to select the S method as the more relevant method to generate modifications of lung parenchyma leading to impaired mechanical properties of the lung needed to simulate the decreased lung compliance observed in IPF. Lastly, as the aim of this study was to simulate a moderate IPF, it was decided to focus on stage 3 (*i.e*. physical method applied to entire upper lobes + bases of lower lobes). Indeed, this stage induces a slight modification of resistances for an important modification of compliance (Figs. S2–S4 in Supplementary Information). The selected method did not include mucus accumulation in the airways, which is an uncommon feature of IPF. Moreover, even if we were unable to precisely assess the mucus status in the broncho-alveolar regions, we could assume that mucus is present and could be accumulated in these regions as observed in IPF-suffering patients^3^.

### Assessment of respiratory mechanics

#### Overall results

Table 1 reports mean ± standard deviation values and 95% confidence interval of experimental data collected for the 30 *ex vivo* respiratory models. As expected, inspiratory time (IT), expiratory time (ET) and respiratory rates (RR) are very close from aimed values meaning that the developed respiratory model is tunable at will and reliable for these RR. Reliability and reproducibility data are presented in Tables S3 and S4 in the Supplementary Information. For “healthy” lungs, two RR were tested: 15 and 25 cycles/min. For “fibrotic” lungs, only 25 cycles/min was used. Fifteen cycles per minute was used as RR for healthy lungs in accordance with our previous data used to validate the “healthy” *ex vivo* respiratory model^41,42,49^. The RR of 25 cycles per minute was chosen to mimic IPF. These RR are similar to those found for healthy adult subjects and IPF-suffering adult patients^8,10,12^. According to experimental data and predefined thresholds – *i.e*. inter-individual variations about 30% and intra-individual variations about 15% - we assumed the reliability and the reproducibility of our model.

**Table 1:** Comparison of respiratory parameters and breathing pattern for each respiratory rate (n = 30; data are presented as mean ± standard deviation [confidence interval 95%]).

	Healthy *ex vivo* model RR set as 15	Healthy *ex vivo* model RR set as 25	Healthy *in vivo* data	IPF *ex vivo* model RR set as 25	IPF *in vivo* data
Aimed RR (cycles/min)	15	25	15*^,^^10^	25	25*^,^^10^
Measured RR (cycles/min)	15.25 ± 0.49 [15.07; 15.43]	25.23 ± 0.82 [24.93; 25.53]	N/A	25.31 ± 0.89 [24.98; 25.64]	N/A
IT (s)	1.42 ± 0.09 [1.39; 1.46]	0.90 ± 0.07 [0.88; 0.93]	1.6*^,^^10^	0.85 ± 0.06 [0.83; 0.87]	0.9*^,^^10^
ET (s)	2.52 ± 0.11 [2.47; 2.56]	1.48 ± 0.07 [1.45; 1.51]	2.4*^,^^10^	1.52. ± 0.08 [1.50; 1.55]	around 1.5*^,^^10^
TV (mL)	513.8 ± 76.8 [485.2; 542.5]	297.1 ± 41.5 [281.6; 312.6]	500^10^	261.1 ± 44.3 [244.5; 277.6]	400*^,^^10^
MV (L/min)	7.84 ± 1.12 [7.42; 8.26]	7.49 ± 1.01 [7.11; 7.86]	7.5*^,^^10^	6.59 ± 1.10 [6.18; 7.00]	10*^,^^10^
MIF (mL/s)	360.6 ± 39.46 [345.9; 375.3]	328.8 ± 30.3 [317.5; 340.1]	400^10^ 440^43^	305.8 ± 39.4 [291.1; 320.5]	400^10^ 440^43^
R (cmH_2_O/(L/s))	13.254 ± 3.29 [12.31; 14.76]	11.68 ± 1.52 [11.11; 12.25]	3.23 ± 0.29^#,^^60^ 2.25 ± 0.28^61^	10.71 ± 1.07 [10.31; 11.11]	N/A
C (mL/cmH_2_O)	90.16 ± 27.72 [79.81; 100.50]	96.28 ± 28.69 [85.57; 107.00]	167,22^#,^^62^	69.39 ± 20.52 [61.73; 77.05]	50*^,^^7^^,^^10^

Respiratory rate (RR), inspiratory time (IT), expiratory time (ET), tidal volume (TV), minute ventilation (MV), mean inspiratory flow (MIF), resistances (R) and compliance (C). N/A: not applicable. *corresponds to value extracted from a plot. #corresponds to data recalculated from correspondent literature.

#### The healthy *ex vivo* respiratory model

Before application of S, tidal volume (TV) was 515.2 ± 77.0 mL and minute-ventilation (MV) was 7.86 ± 1.11 L/min. Mean inspiratory flow (MIF) was 361.4 ± 39.3 mL/s. Lastly, R values were 13.23 ± 3.41 cmH_2_O/(L/s), while dynamic compliance (C) values were 90.44 ± 27.97 mL/cmH_2_O. Observed results are consistent with previously published data concerning the healthy *ex vivo* respiratory model^41,49^ and in good accordance with data available in the literature from *in vivo* studies on healthy subjects^10^. MIF values appear to be similar to values obtained of healthy subjects as showed Renzi *et al*.^10^. Lastly, experimental C values, 90.44 ± 27.97 mL/cmH_2_O, are almost 2 times lower than commonly considered as normal for healthy subjects. The relatively low values obtained with our model were most probably due to the lack of perfusion and the unknown status of surfactant.

#### The fibrotic *ex vivo* respiratory model

We applied the S method to the lungs to reduce lung compliance by modifying the mechanical properties of lungs parenchyma. We also modified the RR, which was set at 25, to better simulate the breathing pattern of an IPF-suffering patient. TV was 259.4 ± 48.8 mL while MV was 6.62 ± 1.10 L/min. Results found in literature showed that TV of IPF-suffering patient is around 400mL^8,10,12^, which is higher than the developed model. R and C values were respectively 10.54 ± 1.39cmH_2_O/(L/s) and 70.27 ± 21.65 mL/cmH_2_O. C values appeared to be relatively close to human *in vivo* data. Indeed, Renzi^10^ *et al*. found values about 43,3 mL/cmH_2_O and Zapletal^7^ *et al*. found a mean value of 50 mL/cmH_2_O.

#### Physiological reliability of the respiratory *ex vivo* model

To sum up, all the 5 respiratory features assessed were found to be in the same order of magnitude as *in vivo* data for adult suffering of IPF and with previously obtained results on “healthy” *ex vivo model*. However, features by features, we observed that some results were different than expected values. Nevertheless, differences observed between our experimental data - particularly the low TV for the IPF model - and those obtained from literature could be due to patients’ ability to increase muscle workload during inspiration and expiration to compensate lung stiffness, while we only applied fixed negative pressures. To compensate this phenomenon, we are currently developing a new depression generator allowing to tune the applied negative pressure in the enclosure. Moreover, some limitations of the *ex vivo* model could impact these respiratory physiological features as the lack of lung surfactant and the absence of blood perfusion. Indeed, surfactant is responsible for approximately 50% of compliance of the system^50^. Perfusion of lungs is also an important parameter related to resistances.

### Assessment of regional gas-ventilation by ^81m^Kr scintigraphies

**Figure 2:**
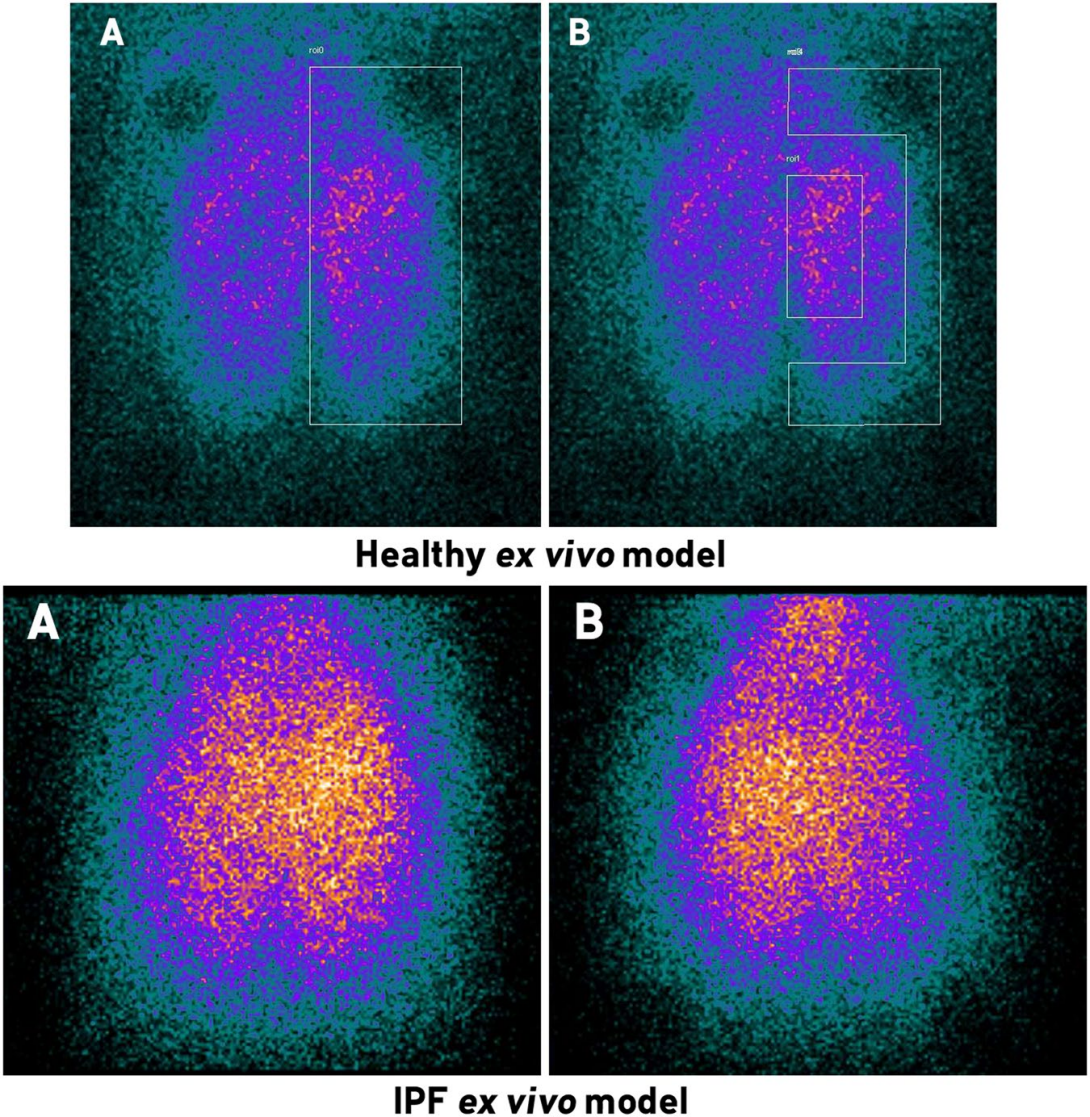
81mkrypton (^81m^Kr) scintigraphic images of IPF simulating lungs. A: anterior view. B: posterior view.

Figure 2 presents scintigraphic images of ^81m^Kr gas-ventilation. Scintigraphic measurements of ventilation using ^81m^Kr were made on 12 lungs whose compliance was modified with the S method at stage 3 (*i.e*. physical method applied to entire upper lobes + bases of lower lobes). Due to the very short half-life of ^81m^Kr, repartition of this gas within lungs is widely considered to allow imaging of regional gas-ventilation^51^. Experiments showed a symmetry in left/right regional gas-ventilation with 50.10% ± 3.60% in the left lung and 49.91% ± 3.60 in the right lung. When compared to the previously developed healthy *ex vivo* model^41,49^, there was no significant differences in terms of left/right regional gas-ventilation (Table 2, Fig. 2 and Figs. S5 and S6 in Supplementary Information). However, when taking into account the central-to-peripheral ratio, we observed that the penetration index of ^81m^Kr gas in our IPF model, 53.10 ± 1.98%, is lower compared to the data observed on the previous healthy *ex vivo* model, which exerted a central-to-peripheral ratio about 62.56%^41^ (*p* < 0.0001). However, literature lacks of quantitative data because ^81m^Kr ventilation scintigraphies are usually coupled with ^99m^Tc-albumin scintigraphy, to assess the qualitative ventilation/perfusion mismatch. Hence, comparison with available *in vivo* data was only qualitative. This gas-ventilation homogeneity is in good accordance with *in vivo* data obtained from patients suffering of IPF^14–16,20,44,52^.

**Table 2:** Left/right ventilation distribution and central/peripheral repartition within lung assessed with ^81m^krypton (^81m^Kr).

	IPF *ex vivo* model		Healthy *ex vivo* model^41,49^	
	Left lung	Right lung	Left lung	Right lung
^81m^Kr repartition (% of total radioactivity)	50.10 ± 3.60 [47.81; 52.38]	49.91 ± 3.60 [47.62; 52.19]	50.31 ± 3.57 [48.33; 52.29]	49.69 ± 3.57 [47.71; 51.67]
Central/peripheral ratio (% of radioactivity in lung)	53.10 ± 1.98 [51.84; 54.36]		62.56 ± 6.24 [58.96; 66.16]	

Data are presented as mean ± standard deviation [95% confidence interval] (n = 12).

### Regional aerosol deposition

#### Healthy versus fibrotic *ex vivo* respiratory model: impact of the alteration of lung compliance on the aerosol regional deposition

**Figure 3:**
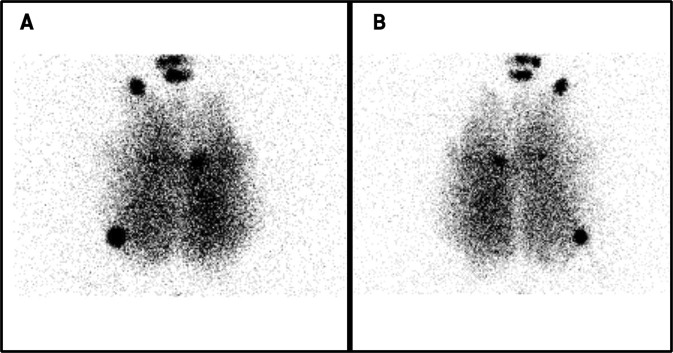
99mtechnetium diethylene triamine pentaacetic acid (^99m^Tc-DTPA) planar images of the *ex vivo* model developed in this study (n = 6). (**A**) anterior view. (**B**) posterior view.

The regional aerosol deposition was recorded by planar scintigraphies using gamma-camera imaging (using 6 different respiratory tracts) and 3D scintigraphic images using SPECT-CT (*n* = 1). Deposited fractions along nebulization system and within respiratory tracts were quantified and results are shown in Table 3. The nebulized fraction corresponds to the proportion of the nominal dose that was efficiently nebulized (*i.e*. the initial activity injected into the nebulizer tank minus the remaining activity that remains after the nebulization process in the device). We observed on 2D scintigraphies (Fig. 3) that radiolabeled aerosol was deposited within lungs with a more intense signal in the central regions of the lungs.

**Table 3:** Deposited fractions along nebulization system as a proportion of nominal dose of radioactivity (mean ± standard deviation, n = 6).

Nebulized fraction	Interface	ENT	Lungs	Filters
10.60 ± 6.15%	1.34 ± 0.86%	1.26% ± 0.61%	5.60% ± 3.27%	2.39% ± 1.81%

Nebulized fraction corresponds to the dose of ^99m^Tc-DTPA that was efficiently nebulized. Interface: naso-buccal mask. ENT: 3D-printed Ears-Nose-Throat replica with connecting tube. Filters: filters connected between depression generator and the enclosure to receive aerosol leaking from the lungs.

**Figure 4:**
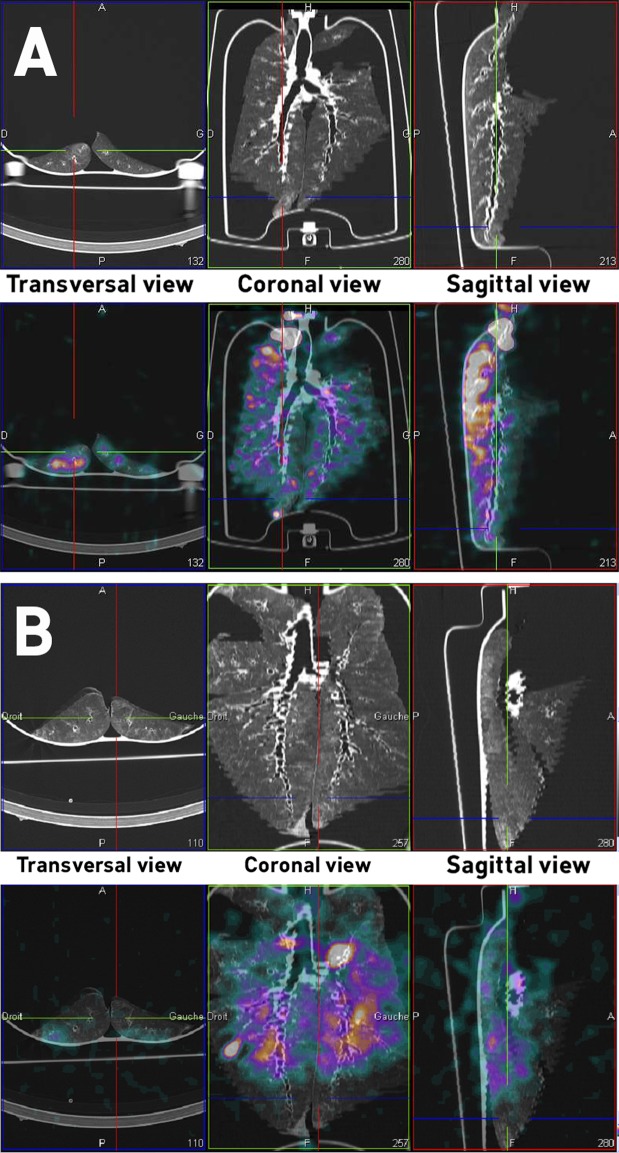
99mtechnetium diethylene triamine pentaacetic acid (^99m^Tc-DTPA) images after three-dimension reconstruction of the *ex vivo* model developed in this study. (**A**) Images from healthy model. (**B**) Images from IPF-mimicking lungs. Upper panels: tomography images. Lower panels: fusion of tomography and scintigraphic images.

Finally, Fig. 4A illustrates the SPECT-CT aerosol deposition imaging within the healthy *ex vivo* model. As easily seen, the deposition pattern is quite homogeneous and the aerosol had a deep penetration in the healthy lung, to reach peripheral regions. At the contrary, Fig. 4B depicts a SPECT-CT image of a fibrotic lung after thermal treatment of the parenchyma with the S method at stage 3. It shows very poor deposition pattern in the bases of lower lobes.

Compared to data generated on the “healthy” *ex vivo* model^41^, aerosol penetration within the fibrotic lung was not as deep. Indeed, lung bases did not show the deposition of the radiolabeled aerosol. These two images showed that despites its limitations, this model presented regional aerosol deposition features associated with reduced lung compliance in regions treated to modify the mechanical properties of the parenchyma. However, it should be noted that the model only allowed to assess regional deposition of the aerosol. The absorption of the ^99m^Tc-DTPA and its diffusion in the different cell types composing parenchyma and mesenchyme of the respiratory tract could not be assessed. The development of a more complex model with perfusion and ventilation is currently under development and could furtherly answer to this limitation.

In a comparative manner, the study from Diaz *et al*.^29^ focused on the delivery and the safety of inhaled interferon-γ. These experiments conducted on human were performed with a different nebulization technology (vibrating mesh nebulizer associated to inhalation-triggered nebulization), which generated an aerosol with larger particles while inhalations were performed with a deep and slow pattern. Therefore, comparison to this study should be carefully made. As seen in Table 4, values from Diaz *et al*. are similar from those obtained with our model, when values are expressed as percentage of the nebulized dose, meaning that the regional aerosol deposition patterns are relatively close.

**Table 4:** Deposited fractions in each components of the developed models.

Model	MMAD	Extra-thoracic regions	Inspiratory part	Expiratory part	Lungs
Diaz *et al*.^29^	1.7 µm	12.6 ± 3.0%	22%*	N/A	65.4 ± 4.8%
Montigaud *et al*.	0.86 µm	15 ± 5%	19 ± 15%	N/A	66 ± 18%

Data are expressed as mean ± standard deviation of the nebulized dose of radioactivity. N/A: not applicable. Extra thoracic regions: 3D-printed replica or endotracheal tube. Inspiratory part: naso-buccal mask. Expiratory part: expiratory filter. MMAD: median mass aerodynamic diameter. *value calculated from available data.

Overall, the presented results showed that aerosol deposition was mainly observed in the non-treated zones of the lungs. This feature is comparable to the one observed in patients. While this particular point seemed to be controversial for inhaled therapies in IPF, it could be a compelling point in favor of such therapies because the healthy regions of the parenchyma could already present molecular abnormalities associated with the proliferation of the fibrotic lesions^21^. Thus, even if it is important to develop medical devices that will allow higher depositions into the “fibrotic” regions, it remains important to also treat healthy regions of the lungs.

#### Reliability of the developed *ex vivo* fibrotic lungs compared to IPF-suffering patients in terms of aerosol regional deposition

Literature of such regional aerosol deposition studies *in vivo* is very scarce and do not allow quantitative comparison to assess deposition patterns. Thus, only qualitative comparisons between present experimental results and data found in literature could be made. However, qualitatively, by comparing our results to *in vivo* human data of IPF-suffering patients using similar nuclear imaging technique and administration procedure, we support the conclusion that the *ex vivo* fibrotic respiratory model is in good accordance with existing *in vivo* inputs of IPF-suffering patients^14,20,31,44,53,54^.

This model raises some limitations, such as the supine position during nebulization process that decreases penetration of airborne particles such as nebulization. As previously observed on a panel of nebulizers with different size distribution^42^, the largest particles – with a MMAD of 2.8 µm - are mostly affected by impact while the smallest particles – with a MMAD of 0.23 µm – are mostly affected by turbulence-driven diffusion and would not be significantly impacted by the supine position. Only intermediate particles – with a MMAD of 0.55 µm – would be affected by the supine position, as the main mechanism driving their deposition is sedimentation. Hotspots are visible on planar scintigraphies due to parenchymal wound on the lungs, creating areas with less resistances and thus generating leakage of radioaerosol from the lungs toward inside this enclosure. However, a filter is placed in between the depression generator and the enclosure allowing to pick up aerosol.

## Conclusion

Despite the release of new treatments, idiopathic pulmonary fibrosis is a devastating pulmonary disease with a poor survival rate. Thus, new therapies are needed to manage this disease and improve patients’ quality of life. Inhaled therapies could be promising treatments for IPF. Indeed, pulmonary delivery could lead to less adverse events for equal or better efficiency. However, due to ethical considerations, *in vivo* aerosol regional deposition studies with IPF-suffering patients are scarce. Since the pulmonary route remains challenging due to the decreased lung compliance, there is need for a useful tool for practical and inexpensive preclinical studies devoted to the local delivery of drugs to fibrotic lungs.

In this study, an *ex vivo* respiratory model of lung fibrosis was developed to assess aerosol regional deposition. This model consists in a 3D-printed adult upper airways replica connected to a porcine respiratory tract placed in a sealed enclosure. This experimental set-up allow to induce passive ventilation of lungs by inducing negative pressures within the enclosure (applying the same physical principle as intrapleural negative pressures observed in human breathing). Breathing pattern are easily and reproducibly controllable. Two RR were tested (*i.e*. 15 and 25 cycles/min) to experimentally assess 5 respiratory physiological measurements: MIF, TV, MV, R and C. Results obtained demonstrated that the breathing pattern of the IPF *ex vivo* model was varying in the same magnitude compared to *in vivo* data of IPF-suffering patients. Then, ^81m^Kr planar scintigraphies were used to assess homogeneity of gas-ventilation of the developed IPF *ex vivo* model. It proved to be qualitatively comparable to gas-ventilation observed in patients^15,16,20,44,52^. Besides, the homogeneity of the gas-ventilation leads to confirm the absence of any ventilation defect due to procedure used to decrease the pulmonary compliance of the IPF *ex vivo* model. Finally, the assessment of regional aerosol deposition was performed with ^99m^Tc-DTPA planar and 3D SPECT-CT images. Results obtained showed similar qualitative regional aerosol deposition compared to those found for *in vivo* human studies. Thus, we successfully developed an *ex vivo* respiratory model to simulate the alterations of pulmonary mechanics seen in lung fibrosis. However, the developed fibrotic lung model suffers from some limitations, such as the supine position, the lack of lung surfactant and the lack of blood perfusion. Moreover, patients suffering from IPF could overcome their failing pulmonary mechanical properties by increasing respiratory workload, while we only applied fixed negative pressures. These limitations are outweighed by advantages of this *ex vivo* model. First of all, this pathological respiratory model seems to be a good and relevant surrogate model to animal experiments for aerosol regional deposition studies, particularly compared to rodent models^55,56^. Moreover, the *ex vivo* respiratory model is fitting the 3 R guidelines (Refine, Reduce and Replace). Secondly, this model is relatively cost-saving. Indeed, it does not need any animal husbandry nor sacrifice of research animals since we used porcine respiratory tracts, considered as waste, obtained from slaughterhouse which are considered as waste. Finally, the *ex vivo* model is easy to use and could be tuned as wanted. Hence, this work demonstrated that this original *ex vivo* respiratory model could reliably simulate IPF for aerosol regional deposition studies. Indeed, by proposing a model with low ethical restrictions and allowing alternative to animal and *in vitro* experiments, we hope that our work would support the preclinical development of inhaled therapies by providing more relevant and reliable data before clinical trials.

## Materials and Methods

### Materials

A stereolithographied adult replica of upper airways, including larynx, was made by three-dimensional printing technology. This Ear-Nose-Throat (ENT) replica was obtained by reconstruction from CT scan images of a healthy subject. Data were obtained from Saint-Etienne teaching hospital. At arrival each patient received an information note explicitly stating that their date could be used for research purposes. Furthermore, written consent from patient is obtained in the Radiology ward. Lastly, use of such data was supervised by the Ethic Committee of Saint-Etienne hospital. It integrated laryngeal structures to mimic vocal folds resistance. This 3D-printed replica was anatomically, geometrically and aerodynamically validated using several techniques (endoscopy and CT scans) and was previously used for several nasal and pulmonary aerosol deposition studies^41,42,49,57^. Porcine respiratory tracts were obtained just after slaughter (DespiViandes, La Talaudière France), satisfying all French sanitary controls, and were used within 24 hours or frozen at −20 °C depending on availability. At slaughtering time, swine were 6 months old, with 44% of female and 56% of male from three different species: large white, pietrain and landrace (with mainly large white). After removal of viscera, carcasses weighted 90 to 93 kg. Visual controls of wounds and sutures were achieved, and a bronchoscopy was performed to ensure the absence of significant obstruction of proximal bronchi. Lungs were placed in a sealed enclosure and ventilated using a depression generator (SuperDimension^®^, Covidien, Dusseldorf, Germany). Figure S1 shows the set-up and is available in Supplementary information. The amplitude of depression during a respiratory cycle was −11 ± 2 kPa (equivalent −112 ± 20cmH_2_O) with a mean depression inside the enclosure of −9 kPa (−92cmH_2_O). These relatively high negative pressures were two time the expected values, according to West^50^. This is probably due to the lack of perfusion and the unknown status of lung surfactant. Thirty tracts were used for measurements of respiratory mechanics. *Ex vivo* lungs were not used during more than 8 hours. For all experiments, the model was set in supine position.

### Development of physical methods to reduce lung compliance mimicking the mechanical behavior of a fibrotic lung

To develop a method to mimic the reduced compliance of fibrotic lungs, 3 physical procedures were tested. For the first method, depending of the size of lungs, 3 to 5 g of ethyl cyanoacrylate glue (G) were applied on chosen areas of lungs and let out to polymerize at least 5 minutes to rigidify the external surface of the lung. The second method used hot steam jets (S), delivered by a steam cleaner, applied for 2 to 3 minutes on the surface of the lungs to induce heat denaturation of lung proteins. The third procedure was the combination of both glue and steam (GS), which were applied sequentially starting by S method with few minutes pause before using G method. During the application of the method, lungs were not inflated. For each method, 5 different incrementing stages were tested (*n* = 4): 0: Healthy stage (without treatment); and 4 stages where the physical methods were applied on 1: apices of upper lobes; 2: entire upper lobes; 3: entire upper lobes + bases of lower lobes; 4: whole lungs. For each stage, respiratory mechanics measurements described below were performed (see the section below). Endpoint of this development was to determine the best physical method (among glue, steam or glue + steam) inducing the highest decrease in lung compliance with the lowest modification of pulmonary resistances. Values were expressed as relative value of the healthy stage of the corresponding method (*i.e*. lungs without changes of compliance), for each physical method tested (*i.e*. glue, steam, or glue + steam). This expression of results allowed to compare only the impact of the physical methods on pulmonary compliance without potential biases due to the intrinsic variability of replicates.

### Assessment of respiratory mechanics

The enclosure was instrumented thanks to Biopac^®^ system (Biopac, Goleta, CA, USA). Airflow at the ENT replica was followed with a pneumotachograph (TSD 117) while pressures in the enclosure were determined with a differential pressure transducer (TSD160D). Each component was connected to an amplifier (DA100C), connected to the acquisition hardware (MP160). Data were recorded for 4 minutes with AcqKnowledge^®^ 5.0 software. Then, eight experimental values were calculated from airflow and pressure: tidal volume (TV), minute ventilation (MV), respiratory rate (RR), inspiratory time (IT), expiratory time (ET), total time (TT), resistances (R) and compliance (C). Mean inspiratory flow (MIF) was manually calculated using Microsoft Office Excel 365 (Microsoft, Redmond, USA). Breathing cycles were identified using the software, tidal volume was obtained by integrating the airflow, resistances were calculated by the difference between pressure inside and outside the enclosure divided by the airflow, and finally the lung compliance was calculated by dividing the volume variation by the pressure variation. IT was fixed at 1.3 s, while ET was fixed at 2.6 s to obtain a RR of 15 cycles per minute, or IT at 0.8 s and ET at 1.6 s at to reach a RR of 25 cycles per minute. These breathing times led to a I:E ratio of 1:2. For each experiment, at least 40 breathing cycles were analyzed to calculate above-mentioned respiratory features and to determine mean ± standard deviation and confidence interval.

We calculated the coefficient of TV, MV, R and C for each replicate to assess the reliability, with a threshold value was set at 15%. The coefficient of variation of TV, MV, R and C were calculated for the whole replication to assess the reproducibility, with a threshold value set at 30%.

### Ventilation and regional aerosol deposition

The evaluation of regional ventilation was performed on 12 respiratory tracts using ^81m^krypton (^81m^Kr) scintigraphies^58^. At each RR, an acquisition was realized with a naso-buccal mask (Ambu^®^, Bordeaux, France) as interface. For the assessment of regional ventilation, two regions of interest (ROI) were drawn on each ventilation scintigraphy to identify left and right lung as well as central area and peripheral area^59^. The aim of these experiments was to assess the homogeneity of the regional gas-ventilation of the *ex vivo* fibrotic lung model and thus, to confirm the absence of any ventilation defect due to a too strong application of physical methods used to decrease the pulmonary compliance.

Deposition experiments were performed ^99m^technetium complexed with diethylene triamine pentaacetic acid (^99m^Tc-DTPA) as scintigraphic tracer. A 100MBq solution (3 mL) was placed in the Venticis II^®^ jet nebulizer (Curium, Paris, France). Generated aerosol (MMAD of 0.86) reached the adult replica of upper airways through the mean of a naso-buccal mask (Ambu^®^, Bordeaux, France). Here, we aimed to quantitatively assess the aerosol regional deposition in the different components of the *ex vivo* model of pulmonary fibrosis (naso-buccal mask, head replica and lungs) to compare our results to: i) the previously developed healthy *ex vivo* respiratory model^41,42^ (to determine the impact of the alteration of lung compliance on the aerosol regional deposition) and ii) to *in vivo* human data from IPF-suffering patients^20,31,44^ using similar nuclear imaging technique and administration procedure (to determine the reliability of the developed *ex vivo* fibrotic lung model compared to IPF patients in terms of aerosol regional deposition).

Two-dimensional scintigraphies were conducted on six respiratory tracts. Experiments were performed with a variable angle dual detector Single Photon Emission Computed Tomography/Computed Tomography (SPECT/ CT, SYMBIA T2; Siemens, Knoxville, TN) with a matrix of 256 × 256. Low-energy, high-resolution collimator (FWHM 8.3 mm at 10 cm) – tested weekly for uniformity (UFOV 533 mm × 387 mm, CFOV 400 mm × 290 mm) – were used. The inhalation experiments needed to assess the initial radioactive dose in the nebulizer. The dose was calculated from the difference of activity between the full and empty syringe with 1-min anterior/posterior scintigraphic images. After inhalation of the aerosol (until atomization of the nebulizer), each component of the system was imaged with 3-min anterior/posterior exposition: nebulize (with dead volume), expiratory filter, ENT replica and respiratory tract. For each image, a ROI was drawn while 3 external ROIs were delimited to assess background. The count of each part was determined with corrections for background radiation, radioactivity decay and tissue attenuation (correction factor calculated for each component). Results were expressed in terms of the nominal dose of radioactivity loaded in the nebulizer.

After 2D acquisition, a SPECT and CT acquisitions could be carried out on the respiratory tract to cartography the anatomical deposition of the aerosol. This 3D SPECT acquisition the respiratory tract consisting of 64 (2 × 32) 30-sec images was coupled with a CT. Imaging parameters for this tomography were: 130 kV; 90mAs; 1.25 mm slice thickness; 0.9 mm increment; 1.6 mm pitch; rotation time of 1.5 s according to a previously developed protocol^42^. The associated hardware platform (Symbia net, Siemens) was used to review and to reconstruct the obtained images with the 3D Ordered-Subset Expectation Maximization (OSEM) algorithm. Default settings (8 subsets, 5 iterations) were used. Before reconstruction, the 3D Butterworth smoothing filter was applied with following parameters: cutoff: 0.45 cycles/cm; order. 5 The final images consisted of a 128 × 128 image matrix, with a 1.23 zoom and a pixel size of 3.9 mm. During reconstruction, scatter correction was used for SPECT images. It was also used to correct the attenuation for CT images. CT and SPECT images were associated into sagittal, coronal and transverse views. There, one respiratory tract was used for these 3D images.

### Statistical analyses

Results are expressed as mean ± standard deviation (95% confidence interval). For development of the physical methods mimicking fibrosis, a non-Gaussian distribution was assumed due to the number of replicates, Kruskal-Wallis’ test was performed. For respiratory mechanics measurements, Gaussian distribution was assessed with a Shapiro-Wilk and d’Agostino normality tests. If Gaussian distribution was validated, a repeated-measure one-way ANOVA with Tukey’s multiple comparisons *post hoc* test was used. If distribution was not Gaussian, Friedmann’s test with Dunn’s multiple comparisons *post hoc* test was performed.

For gas-ventilation studies, after assessing normality as described above, a paired *t*-test was used to compare left and right lung fraction of total counted radioactivity and central-to-peripheral ratio for each lung. Data were compared to those from the previously developed healthy *ex vivo* model^41^ with an unpaired *t*-test. The tested hypothesis was the existence of significant difference of regional ventilation repartition between healthy and IPF *ex vivo* models. All tests were two-sided and *p* < 0.05 was considered statistically significant. Statistical analyses were performed using GraphPad Prism® 7 (GraphPad Software, La Jolla, CA, USA).

## Supplementary information

Supplementary information

## References

[1] Raghu G (2011). An Official ATS/ERS/JRS/ALAT Statement: Idiopathic Pulmonary Fibrosis: Evidence-based Guidelines for Diagnosis and Management. Am. J. Respir. Crit. Care Med..

[2] Duchemann B (2017). Prevalence and incidence of interstitial lung diseases in a multi-ethnic county of Greater Paris. Eur. Respir. J..

[3] Barratt, S. L., Creamer, A., Hayton, C. & Chaudhuri, N. Idiopathic Pulmonary Fibrosis (IPF): An Overview. *J. Clin. Med*., **7**, (2018). 10.3390/jcm7080201 PMC6111543 30082599

[4] Pérez ERF (2010). Incidence, Prevalence, and Clinical Course of Idiopathic Pulmonary Fibrosis. Chest.

[5] Poletti V (2013). Idiopathic Pulmonary Fibrosis: Diagnosis and Prognostic Evaluation. Respiration.

[6] Collard HR (2003). Changes in Clinical and Physiologic Variables Predict Survival in Idiopathic Pulmonary Fibrosis. Am. J. Respir. Crit. Care Med..

[7] Zapletal A, Houštěk J, Šamánek M, Čopová M, Paul T (1985). Lung function in children and adolescents with idiopathic interstitial pulmonary fibrosis. Pediatr. Pulmonol..

[8] Javaheri S, Sicilian L (1992). Lung function, breathing pattern, and gas exchange in interstitial lung disease. Thorax.

[9] Renzi G, Milic-Emili J, Grassino AE (1982). The pattern of breathing in diffuse lung fibrosis. Bull. Eur. Physiopathol. Respir..

[10] Renzi G, Milic-Emili J, Grassino AE (1986). Breathing Pattern in Sarcoidosis and Idiopathic Pulmonary Fibrosis. Ann. N. Y. Acad. Sci..

[11] Sansores RH (1996). Correlation between pulmonary fibrosis and the lung pressure-volume curve. Lung.

[12] Nava S, Rubini F (1999). Lung and chest wall mechanics in ventilated patients with end stage idiopathic pulmonary fibrosis. Thorax.

[13] Davies D, Crowther JS, MacFarlane A (1975). Idiopathic progressive pulmonary fibrosis. Thorax.

[14] Crystal RG (1976). Idiopathic Pulmonary Fibrosis: Clinical, Histologic, Radiographic, Physiologic, Scintigraphic, Cytologic, and Biochemical Aspects. Ann. Intern. Med..

[15] Eary JF, Fisher MC, Cerqueira MD (1986). Idiopathic pulmonary fibrosis. Another cause of ventilation/perfusion mismatch. Clin. Nucl. Med..

[16] Pochis WT (1990). Idiopathic pulmonary fibrosis. A rare cause of scintigraphic ventilation-perfusion mismatch. Clin. Nucl. Med..

[17] Agustí AGN (1991). Mechanisms of Gas-exchange Impairment in Idiopathic Pulmonary Fibrosis. Am. Rev. Respir. Dis..

[18] King TE (1991). Diagnostic Advances in Idiopathic Pulmonary Fibrosis. CHEST.

[19] Fulmer JD, Roberts WC, von Gal ER, Crystal RG (1977). Small Airways in Idiopathic Pulmonary Fibrosis. J. Clin. Invest..

[20] Strickland NH, Hughes JM, Hart DA, Myers MJ, Lavender JP (1993). Cause of regional ventilation-perfusion mismatching in patients with idiopathic pulmonary fibrosis: a combined CT and scintigraphic study. Am. J. Roentgenol..

[21] Plantier L (2018). Physiology of the lung in idiopathic pulmonary fibrosis. Eur. Respir. Rev..

[22] Baddini-Martinez J (2015). Update on diagnosis and treatment of idiopathic pulmonary fibrosis. J. Bras. Pneumol..

[23] Elmufdi F, Henke CA, Perlman DM, Tomic R, Kim HJ (2015). Novel Mechanisms and Treatment of Idiopathic Pulmonary Fibrosis. Discov. Med..

[24] Collard HR (2017). Improving Survival in Idiopathic Pulmonary Fibrosis: The Race Has Just Begun. CHEST.

[25] Mogulkoc N (2001). Pulmonary Function in Idiopathic Pulmonary Fibrosis and Referral for Lung Transplantation. Am. J. Respir. Crit. Care Med..

[26] Martinez, F. *et al*. *The Clinical Course of Patients with Idiopathic Pulmonary Fibrosis*., **142**, (2005). 10.7326/0003-4819-142-12_part_1-200506210-00005 15968010

[27] Fernandez IE, Heinzelmann K, Verleden S, Eickelberg O (2015). Characteristic Patterns in the Fibrotic Lung. Comparing Idiopathic Pulmonary Fibrosis with Chronic Lung Allograft Dysfunction. Ann. Am. Thorac. Soc..

[28] Antoniou KM, Wuyts W, Wijsenbeek M, Wells AU (2016). Medical Therapy in Idiopathic Pulmonary Fibrosis. Semin. Respir. Crit. Care Med..

[29] Diaz KT (2012). Delivery and Safety of Inhaled Interferon-γ in Idiopathic Pulmonary Fibrosis. J. Aerosol Med. Pulm. Drug Deliv..

[30] Maden CH (2018). Safety, tolerability and pharmacokinetics of GSK3008348, a novel integrin αvβ6 inhibitor, in healthy participants. Eur. J. Clin. Pharmacol..

[31] Kanazawa M (1993). [Assessment of pulmonary aerosol deposition and epithelial permeability in 99mTc-DTPA inhalation scintigram]. Nihon Kyobu Shikkan Gakkai Zasshi.

[32] Schüepp KG (2005). *In Vitro* Determination of the Optimal Particle Size for Nebulized Aerosol Delivery to Infants. J. Aerosol Med..

[33] Geiser M, Kreyling WG (2010). Deposition and biokinetics of inhaled nanoparticles. Part. Fibre Toxicol..

[34] Baskin MI (1990). Regional Deposition of Aerosolized Pentamidine: Effects of Body Position and Breathing Pattern. Ann. Intern. Med..

[35] Oosthuizen MA, Oberholzer HM, Scriba MR, van der Spuy WJ, Pretorius E (2012). Evaluation of the morphological changes in the lungs of BALB/c mice after inhalation of spherical and rod-shaped titanium nanoparticles. Micron.

[36] McKinney W (2012). Pulmonary and cardiovascular responses of rats to inhalation of a commercial antimicrobial spray containing titanium dioxide nanoparticles. Inhal. Toxicol..

[37] *Comparative Biology of the Normal Lung*. (Elsevier, 2015). doi:10.1016/C2012-0-01154-4

[38] Köbrich R, Rudolf G, Stahlhofen W (1994). A Mathematical Model of Mass Deposition in Man. Ann. Occup. Hyg..

[39] Kelly JT, Kimbell JS, Asgharian B (2001). Deposition of fine and coarse aerosols in a rat nasal mold. Inhal. Toxicol..

[40] Phalen RF, Oldham MJ (2001). Methods for modeling particle deposition as a function of age. Respir. Physiol..

[41] Perinel, S. *et al*. Development of an *ex vivo* human-porcine respiratory model for preclinical studies. *Sci. Rep*., **7**, (2017). 10.1038/srep43121 PMC5324051 28233793

[42] Perinel, S. *et al*. Micron-sized and submicron-sized aerosol deposition in a new *ex vivo* preclinical model. *Respir. Res*., **17**, (2016). 10.1186/s12931-016-0395-7 PMC4937580 27388488

[43] Olukogbon, K. L., Thomas, P., Colasanti, R., Hope-Gill, B. & Williams, E. M. Breathing pattern and breathlessness in idiopathic pulmonary fibrosis: An observational study. *Respirology*, **21**, 344–349 10.1111/resp.12686 26597757

[44] Watanabe N, Inoue T, Tomioka S, Yamaji T, Endo K (1995). Discordant findings between krypton-81m gas and Tc-99m labeled ultrafine aerosol lung ventilation SPECT in two patients with idiopathic pulmonary fibrosis. Clin. Nucl. Med..

[45] Yeh SH, Liu RS, Wu LC, Peng NJ, Lu JY (1995). 99Tcm-HMPAO and 99Tcm-DTPA radioaerosol clearance measurements in idiopathic pulmonary fibrosis. Nucl. Med. Commun..

[46] Viidik A (1979). Connective tissues — possible implications of the temporal changes for the aging process. Mech. Ageing Dev..

[47] Lacin T (2007). Safety of a thermal vessel sealer on main pulmonary vessels. Eur. J. Cardiothorac. Surg..

[48] Aldous IG, Veres SP, Jahangir A, Lee JM (2009). Differences in collagen cross-linking between the four valves of the bovine heart: a possible role in adaptation to mechanical fatigue. Am. J. Physiol.-Heart Circ. Physiol..

[49] Ragey SP (2015). A human-like *ex vivo* preclinical model for aerosol deposition studies. Eur. Respir. J..

[50] West, J. B. & West, J. B. *Pulmonary pathophysiology: the essentials*. (Wolters Kluwer/Lippincott Williams & Wilkins Health, 2012).

[51] Bajc M (2009). EANM guidelines for ventilation/perfusion scintigraphy. Eur. J. Nucl. Med. Mol. Imaging.

[52] James JM (1992). 99TcmTechnegas and krypton-81m ventilation scintigraphy: a comparison in known respiratory disease. Br. J. Radiol..

[53] Agusti C (1994). Clinical and functional assessment of patients with idiopathic pulmonary fibrosis: results of a 3 year follow-up. Eur. Respir. J..

[54] Mura M (2004). Does technetium-99m diethylenetriaminepentaacetate clearance predict the clinical course of idiopathic pulmonary fibrosis?. Can. Respir. J..

[55] Srour N, Thébaud B (2015). Mesenchymal Stromal Cells in Animal Bleomycin Pulmonary Fibrosis Models: A Systematic Review. Stem Cells Transl. Med..

[56] Jenkins RG (2017). An Official American Thoracic Society Workshop Report: Use of Animal Models for the Preclinical Assessment of Potential Therapies for Pulmonary Fibrosis. Am. J. Respir. Cell Mol. Biol..

[57] Perinel S (2018). Deposition pattern of aerosolized Legionella using an *ex vivo* human-porcine respiratory model. Int. J. Hyg. Environ. Health.

[58] Fazio F, Jones T (1975). Assessment of regional ventilation by continuous inhalation of radioactive krypton-81m. Br. Med. J..

[59] Newman S (2012). Standardization of Techniques for Using Planar (2D) Imaging for Aerosol Deposition Assessment of Orally Inhaled Products. J. Aerosol Med. Pulm. Drug Deliv..

[60] Rissler, J. *et al*. Deposition efficiency of inhaled particles (15-5000 nm) related to breathing pattern and lung function: an experimental study in healthy children and adults. *Part. Fibre Toxicol*., **14**, (2017). 10.1186/s12989-017-0190-8 PMC5385003 28388961

[61] Gobbi A (2013). Short-term variability in respiratory impedance and effect of deep breath in asthmatic and healthy subjects with airway smooth muscle activation and unloading. J. Appl. Physiol..

[62] Spahija JA, Grassino A (1996). Effects of pursed-lips breathing and expiratory resistive loading in healthy subjects. J. Appl. Physiol..

