# Supplementary material for: Development of an ex vivo preclinical respiratory model of idiopathic pulmonary fibrosis for aerosol regional studies
Source: Sci Rep. 2019 Nov 29;9:17949. doi: 10.1038/s41598-019-54479-2 (PMC6884587; doi:10.1038/s41598-019-54479-2)
Supplement: Supplementary file 1 — Supplementary information [file 41598_2019_54479_MOESM1_ESM.pdf]

Development of an *ex vivo* preclinical respiratory model of idiopathic pulmonary fibrosis for aerosol regional studies

Yoann Montigaud[1], Sophie Périnel-Ragey[2,3], Laurent Plantier[4], Lara Leclerc[1], Clémence Goy[2,3], Anthony Clotagatide[2,3], Nathalie Prévôt[2,3], Jérémie Pourchez[1]

[1] Mines Saint-Etienne, Univ Lyon, Univ Jean Monnet, INSERM, U 1059 Sainbiose, Centre CIS, F - 42023 Saint-Etienne France.
[2]INSERM U 1059 Sainbiose, Université Jean Monnet, Saint-Etienne, F-42023, France.
[3]CHU Saint-Etienne, Saint-Etienne, F-42055, France.
[4]CEPR/INSERM UMR1100, LabexMabImprove & Service de Pneumologie et Explorations Fonctionnelles Respiratoires, Hôpital Bretonneau, Université François Rabelais, Tours, France.

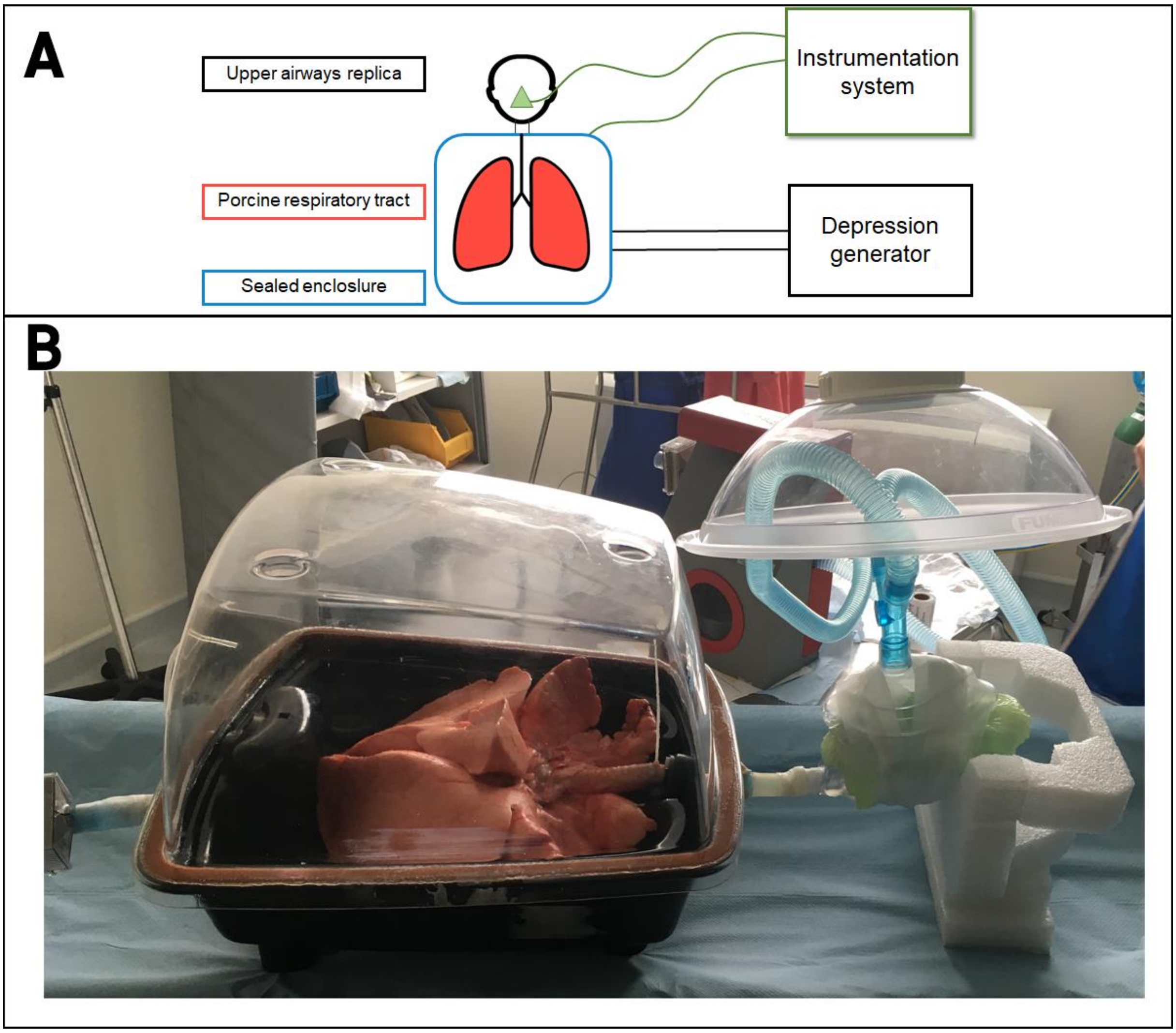

Figure S1 – A: Scheme of the preclininal respirtory model of idiopathic pulmonary fibrosis. B: Photography of the setup in the Nuclear Medicine ward for aerosol deposition study

Results and discussion

Analysis of physical methods to reduce lung compliance mimicking the mechanical behavior of IPF lung

Reliability:

Replicate	Respiratory features	Method	Healthy	Stage 1	Stage 2	Stage 3	Stage 4
1	TV	G	0,70%	0,72%	0,60%	0,74%	0,97%
		S	1,57%	0,90%	0,69%	0,73%	1,12%
		GS	0,68%	0,64%	1,58%	0,66%	1,13%
	MV	G	0,71%	0,70%	0,62%	0,76%	4,35%
		S	1,56%	0,90%	0,72%	0,71%	1,10%
		GS	0,68%	0,64%	1,58%	0,63%	1,14%
	R	G	4,38%	4,19%	82,90%	3,62%	88,29%
		S	6,09%	4,85%	4,19%	4,74%	4,60%
		GS	3,91%	4,09%	8,24%	4,45%	6,05%
	C	G	7,53%	5,46%	3,95%	3,92%	3,53%
		S	10,87%	8,50%	8,71%	7,67%	5,02%
		GS	8,11%	6,50%	6,25%	5,18%	4,10%
2	TV	G	1,51%	0,79%	1,37%	1,10%	2,34%
		S	0,65%	0,98%	0,84%	1,14%	0,86%
		GS	0,72%	0,50%	0,92%	7,25%	4,36%
	MV	G	1,52%	0,79%	1,39%	1,10%	2,33%
		S	0,66%	0,97%	0,85%	1,14%	0,86%
		GS	0,71%	0,56%	0,91%	7,80%	14,92%
	R	G	5,20%	3,63%	93,04%	97,36%	71,39%
		S	4,43%	4,25%	4,77%	4,79%	74,33%
		GS	3,86%	87,13%	71,78%	84,63%	59,73%
	C	G	7,20%	6,60%	6,12%	5,81%	5,04%
		S	8,19%	8,15%	5,53%	5,69%	4,14%
		GS	8,65%	6,64%	7,03%	9,06%	13,29%
3	TV	G	0,95%	0,78%	0,90%	0,76%	1,05%
		S	0,64%	0,77%	0,47%	0,30%	0,40%
		GS	0,79%	21,26%	4,30%	0,65%	0,70%
	MV	G	0,97%	0,79%	0,91%	0,78%	1,05%
		S	0,65%	0,78%	0,48%	0,32%	0,42%
		GS	0,80%	21,26%	4,18%	0,66%	0,71%
	R	G	4,26%	4,72%	4,39%	3,64%	3,40%
		S	4,43%	4,66%	4,08%	3,71%	4,07%
		GS	5,00%	22,66%	15,62%	3,45%	5,75%
	C	G	8,43%	5,66%	5,08%	4,86%	4,74%
		S	8,37%	6,30%	5,30%	5,00%	4,87%
		GS	11,38%	24,82%	6,11%	5,24%	3,58%

Table S1 - Coefficient of variation of tidal volume (TV), minute-ventilation (MV), resistances (R) and compliance (C) for each method and each replicate at each stage. 1: fibrosis of apices of upper lobe; 2: fibrosis of entire upper lobes; 3: fibrosis of entire upper lobes + bases of lower lobes; 4: fibrosis of entire upper lobes + entire lower lobes. "healthy" corresponds to the lungs before modifications, which will be induced at different stages by the corresponding method. Reliability threshold was set at 15%.

Reproducibility:

Method	Respiratory Features	Stages				
		Healthy	1	2	3	4
S	***TV***	5,26%	8,22%	8,16%	7,99%	7,24%
	MV	4,58%	11,83%	13,38%	13,07%	7,71%
	R	5,75%	5,19%	6,39%	8,24%	38,75%
	C	20,68%	21,60%	31,03%	28,19%	8,88%
G	***TV***	5,55%	13,15%	12,83%	11,52%	14,36%
	MV	2,57%	9,69%	9,78%	6,86%	22,97%
	R	5,79%	19,12%	68,90%	47,14%	63,98%
	C	16,39%	26,80%	23,29%	19,79%	24,35%
GS	***TV***	6,80%	4,11%	5,14%	12,60%	7,22%
	MV	7,41%	11,86%	6,91%	7,61%	9,05%
	R	4,41%	43,13%	48,12%	40,77%	42,04%
	C	23,64%	21,28%	17,64%	25,26%	15,74%

Table S2 - Coefficient of variation of tidal volume (TV), minute-ventilation (MV), resistances (R) and compliance (C) of all replicates for each method at each stage. 1: fibrosis of apices of upper lobe; 2: fibrosis of entire upper lobes; 3: fibrosis of entire upper lobes + bases of lower lobes; 4: fibrosis of entire upper lobes + entire lower lobes. "healthy" corresponds to the lungs before modifications, which will be induced at different stages by the corresponding method. Reproducibility threshold was set at 30%.

Statistical analyses

<u>Glue (G) method:</u>

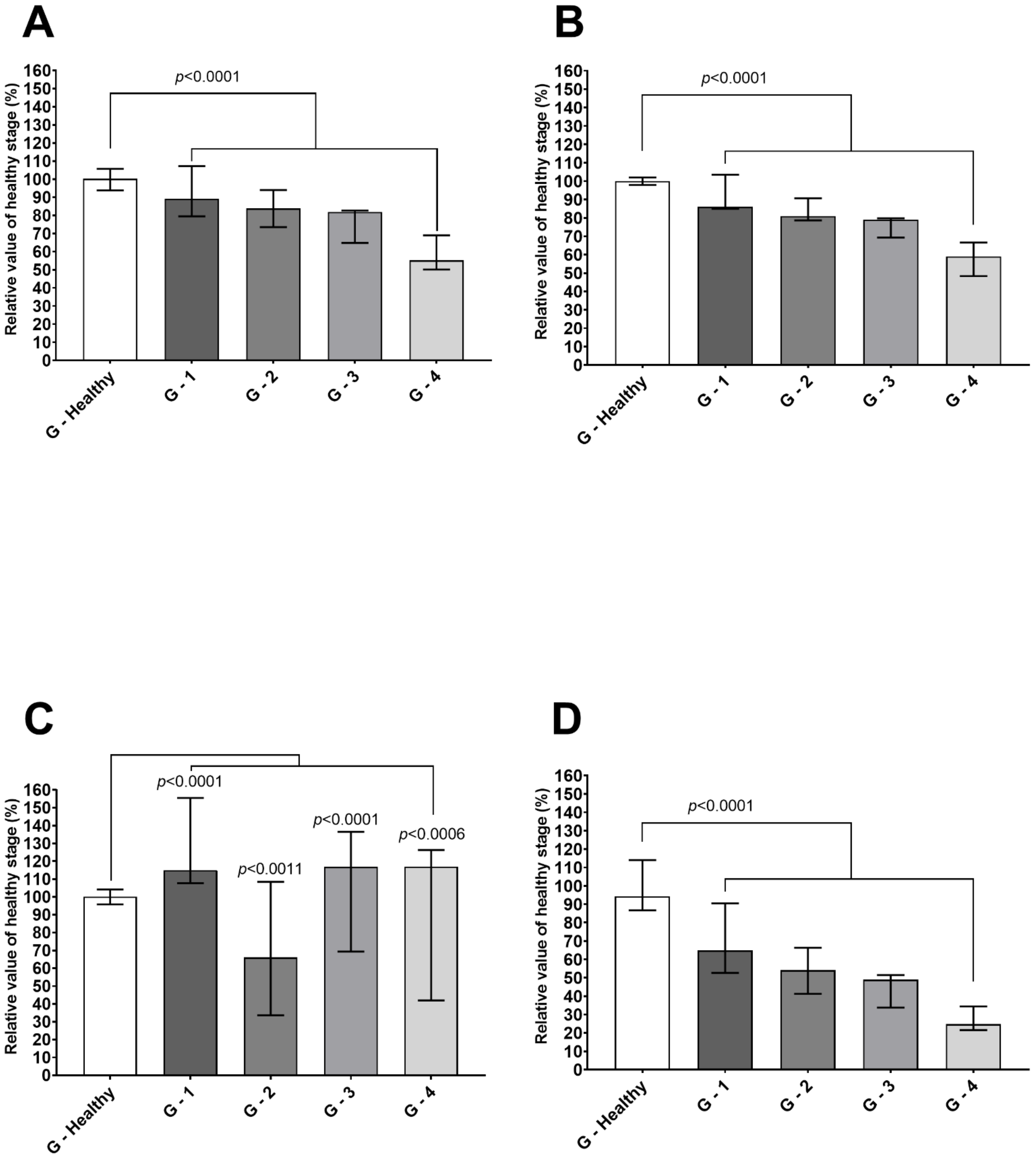

Figure S2 - Variability of tidal volume (A), minute-ventilation (B), resistances (C) and compliance (D) for different stages (n = 4). 1: fibrosis of apices of upper lobe; 2: fibrosis of entire upper lobes; 3: fibrosis of entire upper lobes + bases of lower lobes; 4: fibrosis of entire upper lobes + entire lower lobes. "G healthy" corresponds to the lungs before modifications, which will be induced at different stages by the G method. Data are presented as median and inter-quartile range of the relative value of the healthy stage of the corresponding method

Steam (S) method:

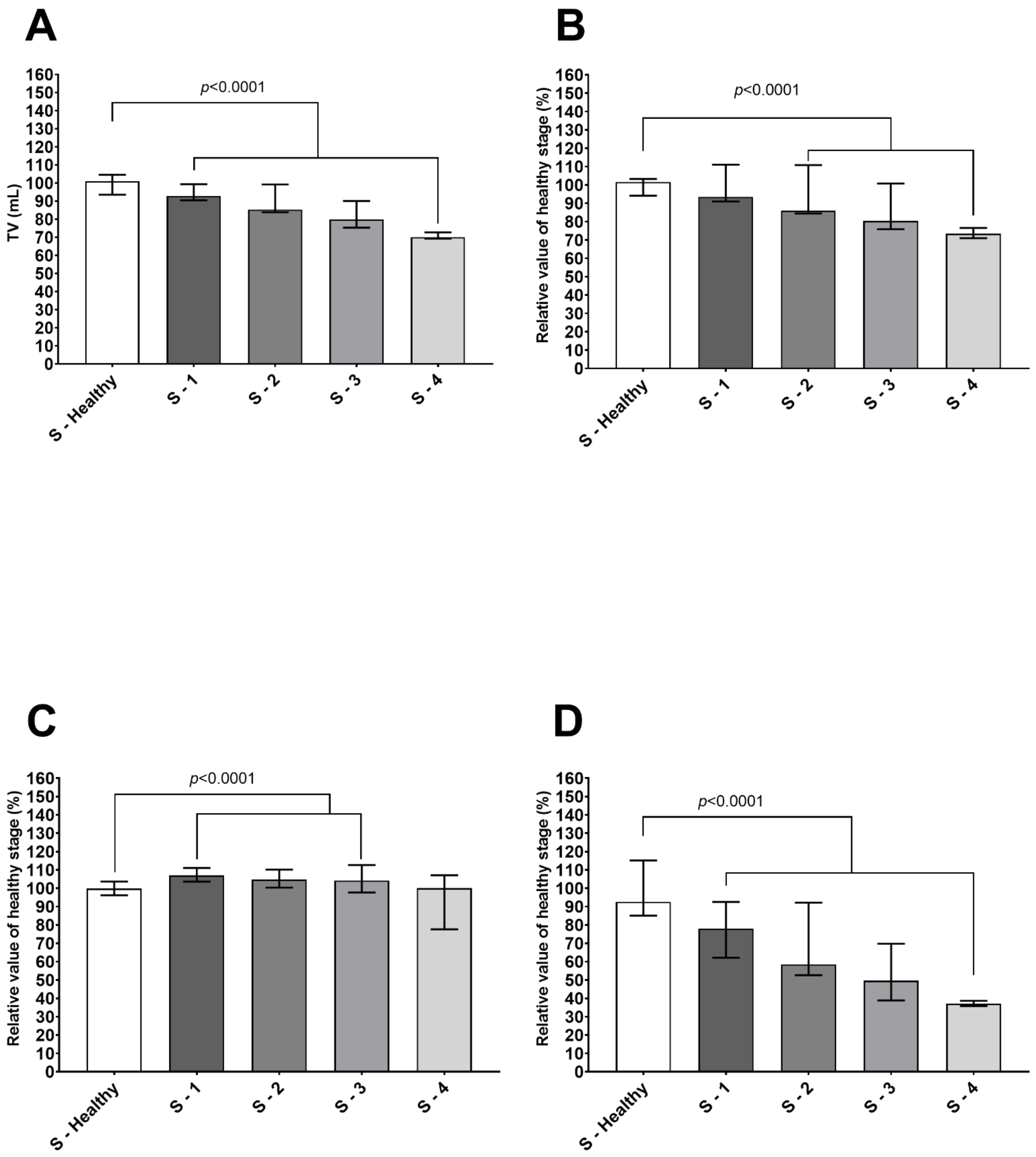

Figure S3 - Variability of tidal volume (A), minute-ventilation (B), resistances (C) and compliance (D) for different stages (n = 4). 1: fibrosis of apices of upper lobe; 2: fibrosis of entire upper lobes; 3: fibrosis of entire upper lobes + bases of lower lobes; 4: fibrosis of entire upper lobes + entire lower lobes. "S healthy" corresponds to the lungs before modifications, which will be induced at different stages by the S method. Data are presented as median and inter-quartile range of the relative value of the healthy stage of the corresponding method

Glue and steam (GS) method:

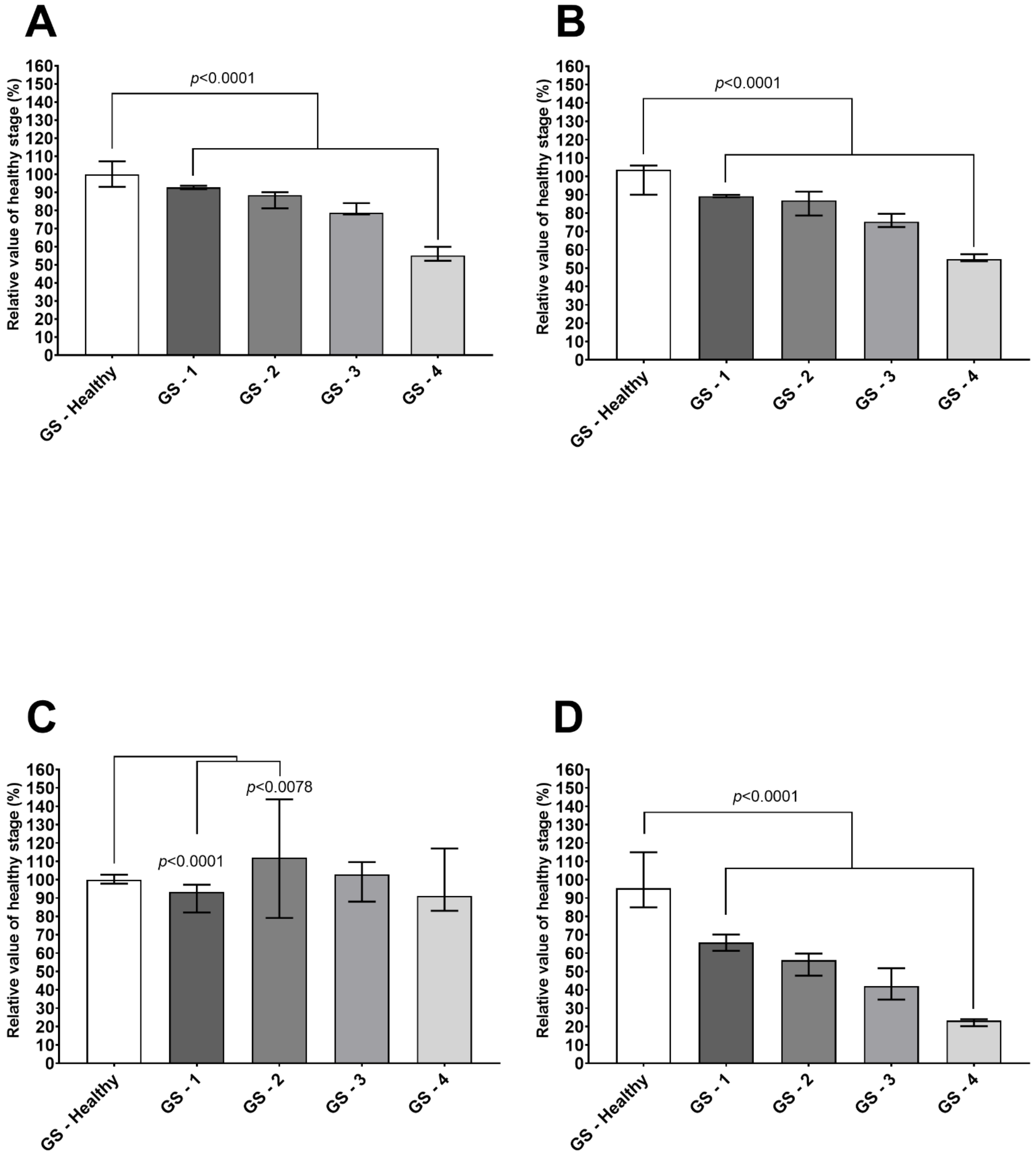

Figure S4 - Variability of tidal volume (A), minute-ventilation (B), resistances (C) and compliance (D) for different stages (n = 4). 1: fibrosis of apices of upper lobe; 2: fibrosis of entire upper lobes; 3: fibrosis of entire upper lobes + bases of lower lobes; 4: fibrosis of entire upper lobes + entire lower lobes. "GS healthy" corresponds to the lungs before modifications, which will be induced at different stages by the GS method. Data are presented as median and inter-quartile range of the relative value of the healthy stage of the corresponding method.

Assessment of respiratory mechanics

Reliability:

	TV			MV			R			C		
**Replicate**	**15**	**25**	**F**	**15**	**25**	**F**	**15**	**25**	**F**	**15**	**25**	**F**
1	3,73%	2,02%	1,89%	3,74%	2,04%	1,90%	3,41%	4,10%	4,12%	5,90%	9,08%	12,27%
2	1,75%	4,16%	3,98%	1,75%	4,17%	3,98%	3,85%	4,00%	4,07%	6,34%	13,39%	10,01%
3	2,35%	2,50%	4,01%	2,35%	2,50%	4,03%	4,32%	4,04%	6,90%	4,92%	10,29%	5,48%
4	3,57%	2,43%	3,23%	3,57%	2,45%	3,23%	6,62%	5,93%	6,99%	7,47%	7,75%	5,34%
5	2,51%	3,16%	2,04%	2,51%	3,17%	2,05%	5,21%	5,04%	3,98%	4,61%	7,91%	6,81%
6	3,66%	1,35%	3,13%	3,65%	1,35%	3,13%	3,02%	4,08%	4,38%	6,64%	5,73%	7,36%
7	5,02%	0,89%	1,85%	5,04%	0,91%	1,85%	4,29%	3,75%	3,96%	14,82%	8,71%	6,15%
8	1,04%	1,77%	1,47%	1,05%	1,78%	1,46%	2,64%	2,82%	3,76%	6,06%	10,27%	9,66%
9	0,73%	1,34%	2,00%	0,73%	1,34%	1,99%	3,33%	3,27%	3,52%	4,33%	11,21%	7,72%
10	3,51%	2,72%	9,36%	3,51%	2,73%	9,37%	3,55%	3,76%	6,10%	10,22%	13,84%	13,31%
11	7,97%	3,84%	4,44%	7,97%	3,84%	4,43%	3,21%	3,83%	4,72%	10,01%	8,71%	12,04%
12	3,79%	2,41%	3,04%	3,81%	2,40%	3,05%	4,75%	3,89%	4,80%	6,77%	6,20%	7,22%
13	3,20%	2,48%	7,21%	3,21%	2,49%	7,21%	4,86%	3,65%	5,95%	6,62%	9,47%	9,89%
14	2,03%	2,90%	8,42%	2,04%	2,89%	9,52%	3,14%	3,82%	8,43%	5,62%	10,93%	12,89%
15	2,84%	5,26%	4,53%	2,82%	5,26%	4,55%	3,92%	4,86%	4,10%	5,32%	10,11%	9,50%
16	2,56%	4,67%	6,52%	2,56%	4,67%	6,52%	4,27%	5,78%	5,88%	7,29%	9,99%	10,65%
17	10,72%	3,42%	6,39%	10,73%	3,42%	6,40%	5,78%	4,53%	7,16%	13,93%	7,50%	10,42%
18	6,82%	3,44%	2,56%	6,83%	3,45%	2,56%	5,22%	5,99%	5,28%	11,42%	12,97%	8,63%
19	2,29%	3,28%	10,36%	2,26%	3,30%	10,36%	5,38%	3,65%	5,77%	14,46%	10,74%	14,59%
20	0,71%	1,96%	7,90%	0,71%	1,96%	7,91%	3,22%	4,02%	5,37%	6,14%	10,76%	14,36%
21	14,65%	8,32%	6,36%	14,65%	8,36%	6,36%	7,46%	6,67%	5,82%	14,20%	14,63%	10,89%
22	1,87%	0,78%	1,49%	1,87%	0,77%	1,51%	3,06%	3,35%	4,56%	5,40%	5,92%	7,03%
23	1,27%	1,12%	1,01%	1,27%	1,10%	0,99%	2,61%	3,36%	3,64%	7,09%	8,47%	7,17%
24	3,02%	0,47%	0,78%	3,02%	0,47%	0,77%	5,68%	3,41%	3,35%	4,61%	5,19%	5,92%
25	1,29%	1,19%	1,93%	1,29%	1,20%	1,93%	2,54%	2,81%	3,40%	3,66%	6,84%	5,18%
26	1,15%	0,88%	1,06%	1,14%	0,88%	1,05%	2,41%	2,89%	3,40%	6,26%	8,29%	6,82%
27	0,94%	3,23%	2,85%	0,93%	3,24%	2,88%	2,91%	3,51%	3,38%	4,26%	8,15%	8,11%
28	0,65%	0,67%	0,61%	0,65%	0,64%	0,60%	3,30%	3,35%	3,45%	2,64%	7,40%	5,95%
29	0,73%	0,59%	0,52%	0,74%	0,60%	0,50%	3,65%	3,30%	4,13%	4,38%	7,08%	6,89%
30	1,52%	0,75%	1,12%	1,52%	0,74%	1,09%	2,74%	3,44%	5,02%	3,02%	5,42%	4,93%
Mean	**3,26%**	**2,47%**	**3,74%**	**3,26%**	**2,47%**	**3,77%**	**4,01%**	**4,03%**	**4,85%**	**7,15%**	**9,10%**	**8,77%**
SD	**2,05%**	**1,26%**	**2,31%**	**2,06%**	**1,26%**	**2,36%**	**1,05%**	**0,72%**	**1,10%**	**2,63%**	**2,02%**	**2,43%**

Table S3 - Coefficient of variation of tidal volume (TV), minute-ventilation (MV), resistances (R) and compliance (C) and each replicate Reliability threshold was set at 15%.

Reproducibility:

TV			MV			R			C		
**15**	**25**	**F**	**15**	**25**	**F**	**15**	**25**	**F**	**15**	**25**	**F**
14,94%	13,97%	16,97%	14,25%	13,49%	16,61%	24,30%	12,99%	9,99%	30,74%	29,80%	29,58%

Table S4 - Coefficient of variation of tidal volume (TV), minute-ventilation (MV), resistances (R) and compliance (C) of all replicates. Reproducibility threshold was set at 30%.

Assessment of regional gas-ventilation by ^{81m}Kr scintigraphies

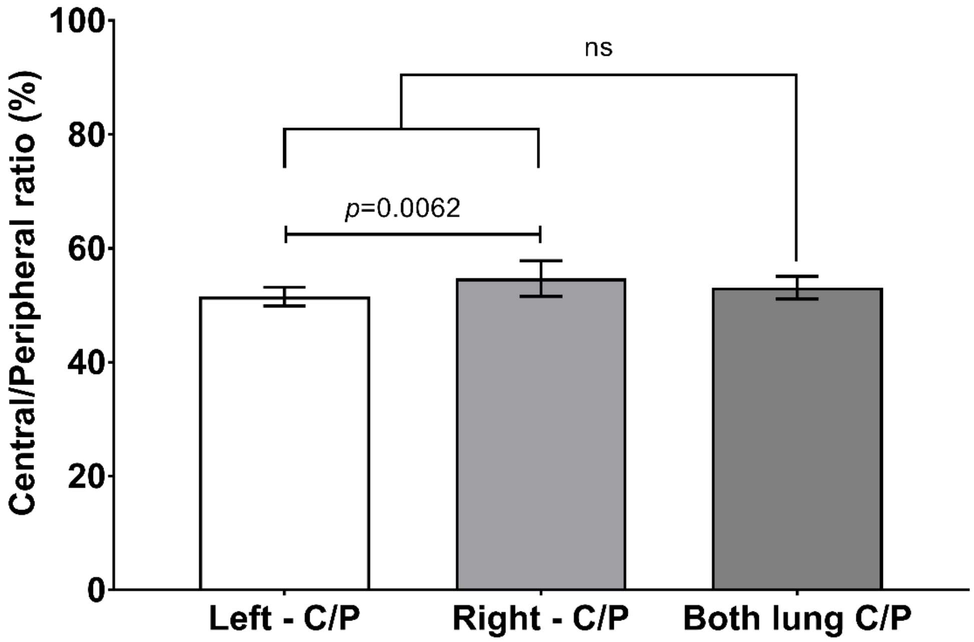

Figure S5 - Central-to-peripheral ratio of each lung and for the whole respiratory tract expressed as mean ± SD of the percentage of nominal dose of radioactivity. C/P: Central-to-peripheral ratio. .

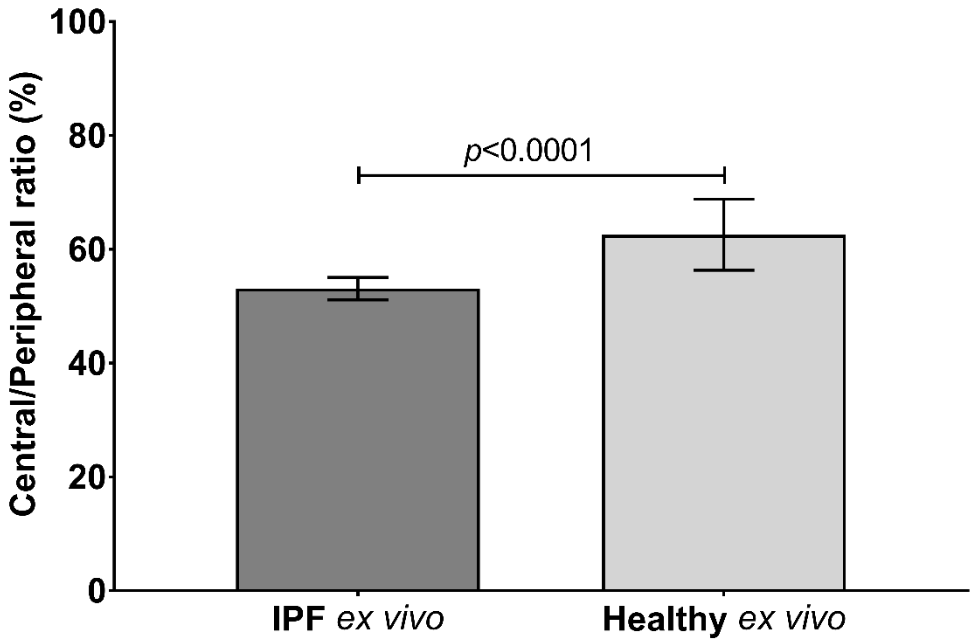

Figure S6 - Central-to-peripheral ratio of healthy and IPF-mimicking ex vivo model. Data are expressed as mean ± SD of the percentage of nominal dose of radioactivity.
